# Exploring the genetic association of allergic diseases with cardiovascular diseases: a bidirectional Mendelian randomization study

**DOI:** 10.3389/fimmu.2023.1175890

**Published:** 2023-06-02

**Authors:** Shilin Wang, Hao Liu, Peiwen Yang, Zhiwen Wang, Poyi Hu, Ping Ye, Jiahong Xia, Shu Chen

**Affiliations:** ^1^ Department of Cardiovascular Surgery, Union Hospital, Tongji Medical College, Huazhong University of Science and Technology, Wuhan, China; ^2^ Department of Cardiology, The Central Hospital of Wuhan, Tongji Medical College, Huazhong University of Science and Technology, Wuhan, China

**Keywords:** asthma, allergic disease, atrial fibrillation, cardiovascular disease, Mendelian randomization

## Abstract

**Background:**

In observational and experimental studies, allergic diseases (AD) have been reported to be associated with some types of cardiovascular diseases (CVD), as both share common pathophysiological processes involving inflammation and metabolic disorders. However, the direction of the causal association between them remains unclear. This Mendelian randomization (MR) study aims to examine the bidirectional causality between AD and CVD.

**Methods:**

We utilized publicly available genome-wide association study (GWAS) summary statistics data from European participants in the UK Biobank and the IEU Open GWAS database. Genetic variants associated with AD, asthma, and CVD were identified and used as instrumental variables to investigate the genetically causal association between them. MR analyses were performed using various analytical methods, including inverse variance weighted-fixed effects (IVW-FE), inverse variance weighted-multiplicative random effects (IVW-RE), MR-Egger, weighted median, weighted mode, and maximum likelihood. Sensitivity tests were conducted to assess the validity of the causality.

**Results:**

The MR analysis with the IVW method revealed a genetically predicted association between AD and essential hypertension [odds ratio (OR)=0.9987, 95% confidence interval (CI): 0.9976-0.9998, P=0.024], as well as between asthma and atrial fibrillation (OR=1.001, 95% CI: 1.0004-1.0017, P=6.43E-05). In the reverse MR analyses, heart failure was associated with allergic diseases (OR=0.0045, 95% CI: 1.1890E-04 - 0.1695, P=0.004), while atherosclerosis (OR=8.7371E-08, 95% CI: 1.8794E-14 - 4.0617E-01, P=0.038) and aortic aneurysm and dissection (OR=1.7367E-07, 95% CI: 3.8390E-14 – 7.8567E-01, P=0.046) might be protective factors of asthma. However, after a Bonferroni correction, only the association between asthma and atrial fibrillation remained robust.

**Conclusion:**

The MR study revealed that asthma is a predominant risk of atrial fibrillation in European individuals, consistent with most experimental and observational studies. Whether AD affects other CVD and the causality between them needs further investigation.

## Introduction

The global prevalence of allergic diseases (AD) has increased dramatically, posing a considerable burden on global health (1). In some countries, nearly a quarter of the population is affected by AD (2). Epidemiological studies have identified risk factors for certain cardiovascular diseases (CVD), including inflammatory and immune responses, which are the primary pathophysiological processes of AD (3, 4). As a result, ADs have emerged as potential risk factors for CVD development, as reported by numerous experimental and observational studies (4–11). These studies revealed that CVD, including atrial fibrillation (5), atherosclerosis (4), heart attack (8), heart failure (8), hypertension (9, 10), stroke (6), and coronary heart disease (11) can be affected by asthma (5–7), atopic dermatitis (5, 8), and rhinitis (5, 9, 10). Nonetheless, the findings of these investigations have been inconclusive. In certain studies, AD has been identified as a potential risk factor for CVD (4–10). On the other hand, contrasting results have been proposed by other researchers (11).

Despite numerous observational studies examining the links between AD and CVD, the presence of confounding factors and unclear causal direction have led to biased conclusions. In an effort to minimize the impact of confounding variables and reverse causation, the Mendelian randomization (MR) approach was developed. In MR, the causal relationship between exposure and outcome are evaluated by instrumental variables (IVs). Genetic variants, which are distributed randomly during meiosis and remain unaltered after conception, are typically employed as IVs to enhance the validity of the findings. To ensure unbiased estimations, genetic variants can serve as IVs only if they fulfill the following criteria (12): First, the variants must exhibit a strong association with exposure. Second, the variants must be independent of any confounding factors related to the exposure-outcome connection. Finally, the variants should influence the outcome solely through the exposure pathway, not *via* any alternative biological routes. Moreover, MR can be extensively applied due to the availability of public genetic data.

Here, a bidirectional MR analysis was performed to investigate the causal relationship between AD and CVD, utilizing publicly accessible summary statistics.

## Methods

### Study design overview

**Figure 1:**
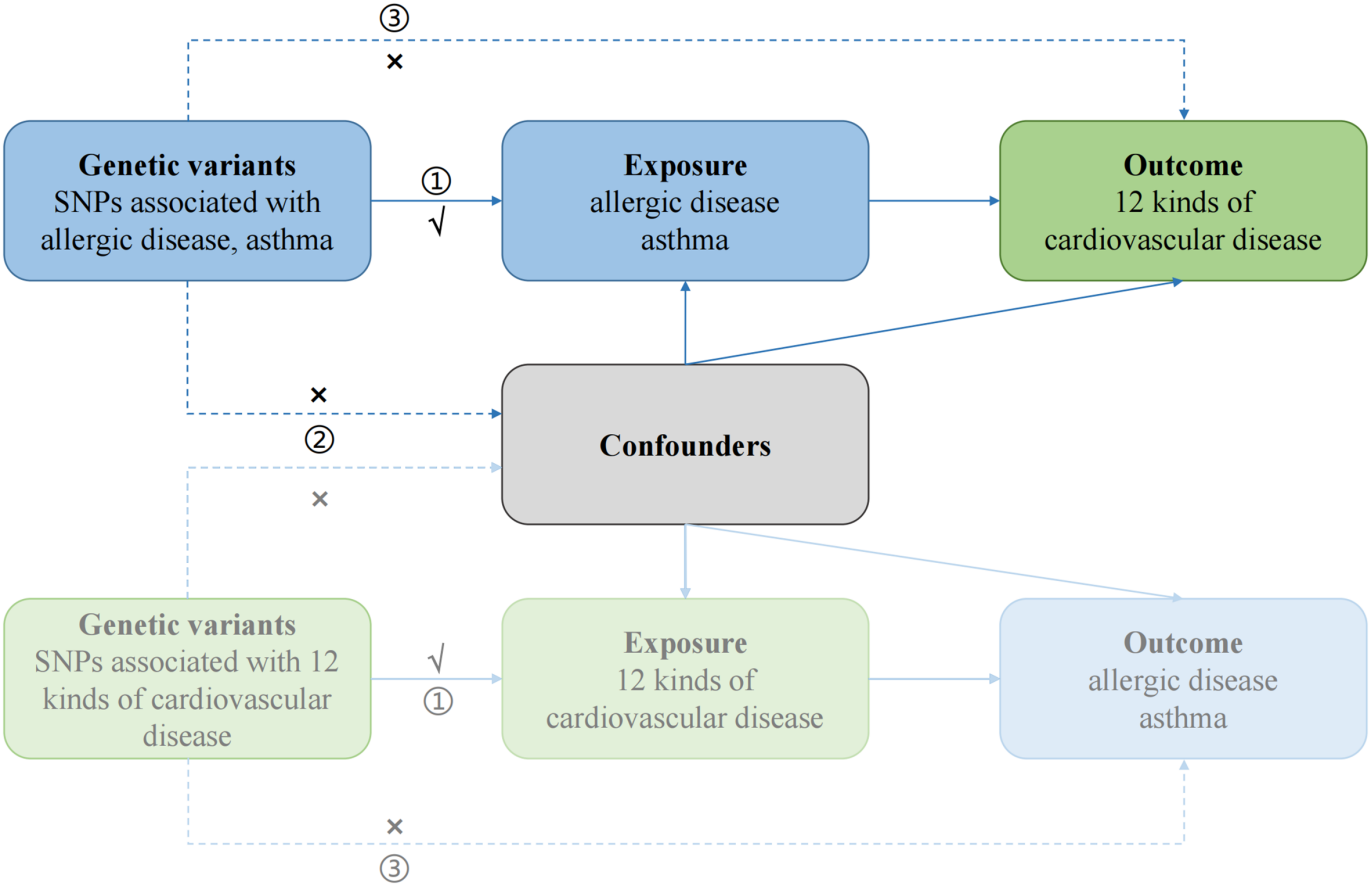
Study design of bidirectional Mendelian randomization study between AD and 12 kinds of CVD. The genetic variants used as instrumental variables meet the three conditions: ① the variants should be highly associated with exposure; ② the variants should be independent of confounding factors of the exposure-outcome association; and ③ the variants affect the outcome only *via* the exposure pathway and not through other biological pathways. The solid paths are significant; the dashed paths should not exist in our study.

First, the causal effects of AD on CVD were estimated, followed by an evaluation of the causal effects of CVD on AD (**Figure 1**). Genetic variants were required to meet three strict assumptions (12). Summary statistic datasets from recent genome-wide association studies (GWAS) of AD, asthma, and CVD were utilized.

### Data sources and SNP selection of genetic instruments for AD

AD comprises asthma, hay fever, and eczema, which share many genetic variants that dysregulate the expression of immune-related genes and often coexist in the same individuals. The summary statistics for AD were obtained from the GWAS catalog (https://www.ebi.ac.uk/gwas/downloads/summary-statistics) or the IEU OpenGWAS project (https://gwas.mrcieu.ac.uk/) conducted by Ferreira et al. (13), which included 360,838 European individuals (180,129 cases *vs* 180,709 controls). Since many observational studies focused only on asthma (5–7) rather than all three ADs, we also estimated the causal effects of asthma on CVD. The summary statistics for asthma were obtained from the GWAS catalog or the IEU OpenGWAS project conducted by Demenais et al., including 127,669 individuals of European ancestry (19,954 cases *vs* 107,715 controls) (14). Detailed information on the conduction procedures and diagnostic criteria is described in the original publications. The sample characteristics of the study population are described in **Supplementary Table S1**.

Genetic variants associated with AD and asthma at genome-wide significance (*P*<5×10^-8^ and 5×10^-6^, respectively) were reported by the GWAS. Simultaneously, a linkage disequilibrium (LD) test was conducted to ensure the independence of clumped SNPs. The LD criteria were established as SNPs with r^2^ > 0.001 and a physical distance of kb< 10,000. SNPs with the lowest P values were retained. Subsequently, all 74 and 39 remaining SNPs related to AD and asthma, respectively, were examined in the Phenoscanner database (http://www.phenoscanner.medschl.cam.ac.uk/) to determine if these SNPs were associated with confounding factors or directly influenced the outcome (15) (*P*<5×10^-8^). We identified 10 SNPs associated with AD and six SNPs associated with asthma that were linked to confounders, as presented in **Supplementary Table S2**. After excluding these SNPs from the AD and asthma GWAS to mitigate potential pleiotropic effects, the remaining SNPs were utilized as IVs in the bidirectional MR analysis (refer to **Supplementary Tables S3, S4**).

### Data sources and SNP selection of genetic instruments for CVD

The second round of GWAS results from the UK Biobank (UKB) (http://www.nealelab.is/uk-biobank) provided us with summary statistics on CVD, which encompassed heart arrhythmia, atrial fibrillation, supraventricular tachycardia, atherosclerosis, aortic aneurysm and dissection, stroke, peripheral vascular disease, cardiomyopathy, heart valve problems, heart failure, myocardial infarction, and essential hypertension. The UKB study is a prospective cohort study that gathered genetic and other data from over 500,000 individuals residing in the UK (16). These diseases were defined and identified by the UKB study based on self-reported status or ICD10 codes. **Supplementary Table S1** summarizes the specifics of these various characteristics, including sample size, SNP count, phenotype code, and others.

We obtained the genome-wide significant variants (P< 5×10^-5^) associated with these CVDs, ensuring their independence (r^2^< 0.001, kb > 10,000). In a similar manner, we examined all SNPs using the Phenoscanner database and removed all SNPs linked to confounding variables, as shown in **Supplementary Table S2**. Details regarding the SNPs utilized as IVs are presented in **Supplementary Tables S5–S16**.

### MR analysis

Following the removal of SNPs whose proxy SNPs were unavailable in the outcome GWAS, we harmonized the exposure and outcome data before evaluation of the association between AD or asthma and CVD. In the meantime, palindromic SNPs with intermediate allele frequency were excluded.

We employed the TwoSampleMR package (17) in R software (version 4.2.2) to conduct bidirectional MR analysis. The Wald ratio method was utilized to calculate the effect of each SNP, and a meta-analysis of the individual effect of each SNP was performed by inverse variance weighting (IVW) to generate the concluding beta estimate (beta _outcome_/beta _exposure_). The inverse variance weighted-fixed effects (IVW-FE) method was our primary analytical approach, as it is the most efficient and widely used. Since the outcome was binary (18), we converted it to the odds ratio (OR). To detect possible violations of IVs assumptions because of directional horizontal pleiotropy, namely the third assumption of SNP, we applied MR-Egger, which can effectively test the null causal hypothesis and serves as the basis method for horizontal pleiotropy in our analysis (19). The IVW method assumes that all SNPs are valid IVs, which can be supplemented by a weighted median (WM) model that provides a consistent effect even if half of the SNPs are pleiotropic (18). All three methods were applied to assess causal robustness of different assumptions, given the introduction of multiple genetic variants (18). Additionally, we employed maximum likelihood methods and weighted mode-based estimates to further analyze the association between exposure and outcome. The maximum likelihood method considers the sample overlap in two-sample MR and the uncertainty of the SNP-exposure association, which is disregarded in IVW (20). The weighted mode-based estimate is resilient to horizontal pleiotropy and exhibits lower type-I error rates than other methods, such as IVW, MR-Egger, WM, and simple median methods (21).

Heterogeneity based on IVW and MR-Egger methods was quantified using Cochran Q statistics and I2 statistics (22). We employed the MR pleiotropy residual sum and outlier (MR-PRESSO) test (23) to detect outlier SNPs (Nb Distribution = 10000, Significant Threshold = 0.05), which was conducted by the package “MR-PRESSO”, and the results are presented in **Supplementary Table S17**. Furthermore, a “leave-one-out” analysis was performed to determine if any single SNP was overly sensitive and disproportionately responsible for the outcome. F-statistics were used to evaluate the strength of SNPs to satisfy the first assumption (17). After determining the effect allele frequency (MAF) of the selected SNPs using the package “LDlinkR”, we calculated the F-statistic with the formula:


$$F=\left(N-k-1\right)/N\times R^{2}/\left(1-R^{2}\right)$$

(N = sample size of the exposure, k = the number of selected SNPs, and R^2^ represents the phenotype variance induced by the SNPs.) When R^2^ is not available, we used the formula:


$$R^{2}=2\times MAF\times \left(1-MAF\right)\times {\left(\beta /SD\right)}^{2}$$

(β= the effect value of the genetic variant of the exposure, MAF = the effect allele frequency of selected SNPs, SD=SE×√N , SE = the standard error of the genetic variant of the exposure, and N = sample size of the exposure). All instruments exhibited F-statistics above the standard cutoff (>10), indicating the presence of sufficiently powerful instruments (24). Each R^2^ and F are shown in **Supplementary Table S17**.

Given the multiple testing, a p-value below 0.002 (0.05/24) was considered robust significance after a Bonferroni correction. A p-value between 0.002-0.05 was considered suggestive significance, and a p-value above 0.05 was considered no significance. All statistical analyses were two-sided.

## Results

### The causal effect of AD and asthma on CVD

In total, 64 and 33 LD-independent genetic variants were utilized as IVs for AD and asthma, respectively, following P value selection and LD clumping (refer to **Supplementary Tables S3, S4**). After removing SNPs that were unavailable in the outcome GWAS and distorted SNPs detected by MR-PRESSO and the “leave-one-out” test, the remaining SNPs were employed as IVs for AD and asthma. The “leave-one-out” test outcomes for the final SNPs are displayed in **Supplementary Figures S1**, **S2**. The F-statistics for the IVs of AD and asthma indicated that the IVs were robust instruments that reduced the bias of IV estimates.

**Figure 2:**
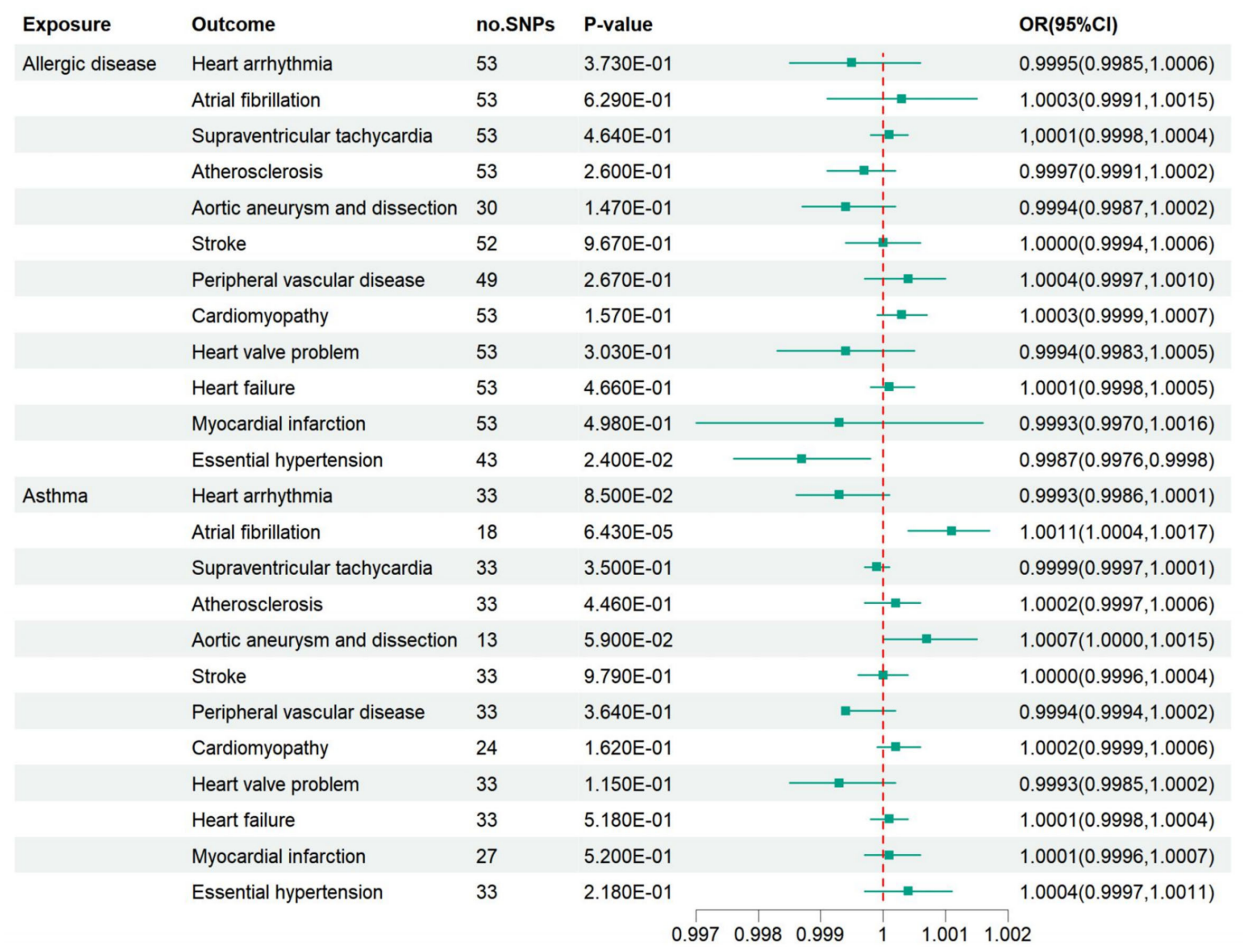
Associations of AD and asthma with 12 kinds of CVD. AD may be a protective factor against essential hypertension, and asthma is suggested to be a risk factor for atrial fibrillation. However, after a Bonferroni correction, there was only a robust association between asthma and atrial fibrillation. OR, odds ratio; CI, confidence interval.

We primarily employed the IVW-FE method to examine the genetic correlation between AD or asthma and CVD. Genetically predicted AD was linked to essential hypertension [odds ratio (OR)=0.9987, 95% confidence interval (CI): 0.9976-0.9998, P=0.024], potentially serving as a protective factor. However, this significance disappeared after applying the Bonferroni correction. Asthma was proposed as a risk factor for atrial fibrillation (OR=1.001, 95% CI: 1.0004-1.0017, P=6.43E-05), with robust significance after the Bonferroni correction, as depicted in **Figure 2**. The outcomes of other models are displayed in **Supplementary Table S18**. Sensitivity analyses were conducted to detect horizontal pleiotropy presence, confirming the IVW results’ reliability, as seen in **Supplementary Table S19**. Cochran’s Q test did not identify any heterogeneity based on IVW and MR-Egger tests, as shown in **Supplementary Table S20**. Reporting bias results, tested by MR-Egger and IVW, are presented in funnel plots (refer to **Supplementary Figures S3, S4**). The confounding factors’ impact on outcomes is illustrated in scatter plots (refer to **Supplementary Figures S5, S6**). Forest plots for each association pair for casualty are provided in **Supplementary Figures S7**, **S8**.

### The causal effect of CVD on AD and asthma

**Figure 3:**
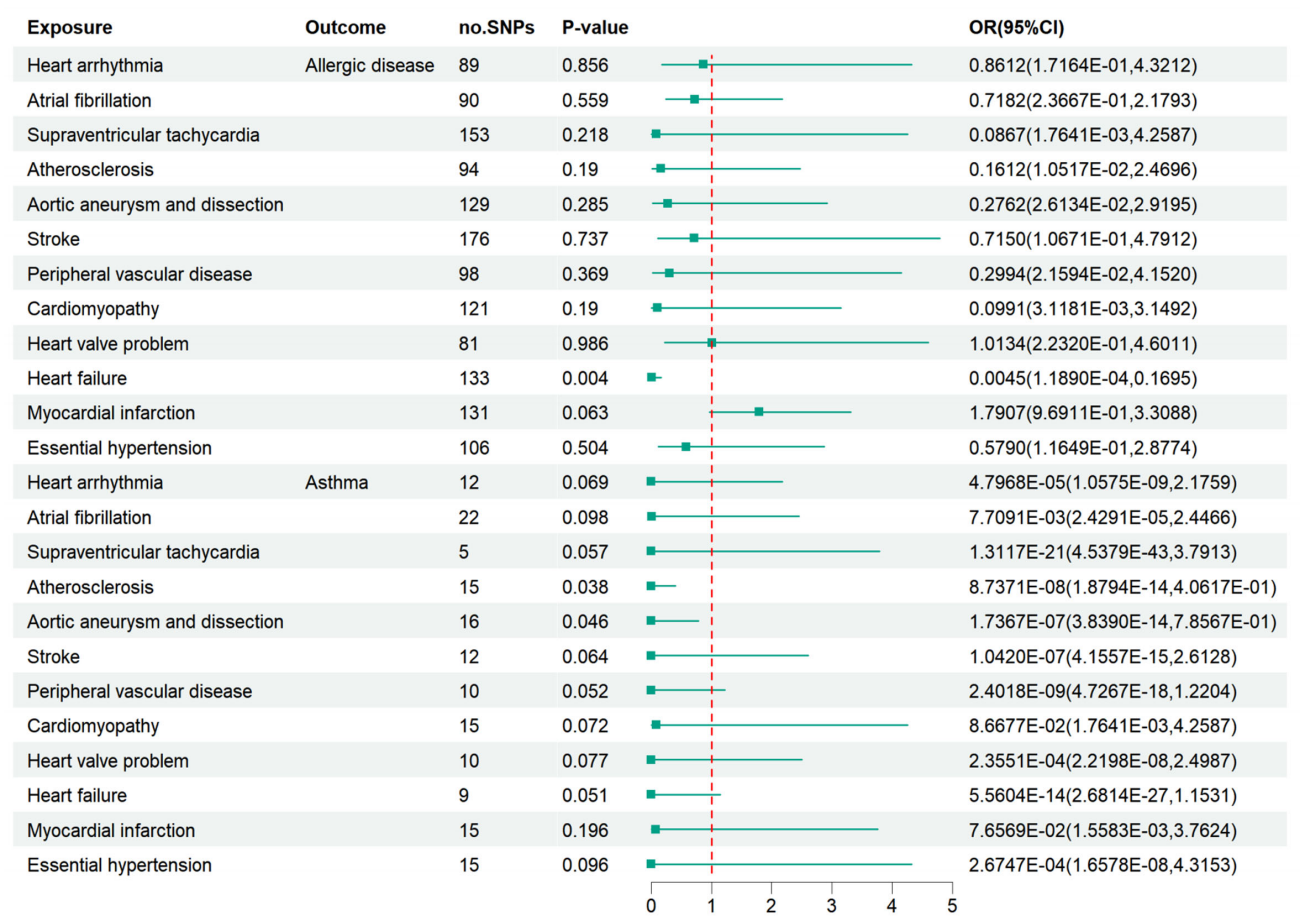
Associations of bioavailable AD and asthma with 12 kinds of CVD. Essential hypertension was suggested to be a protective factor against AD and asthma. Heart failure may be a suggestive protective factor of AD. Atherosclerosis and aortic aneurysm/dissection may be suggestively associated with asthma. However, there was no robust association between cardiovascular disease and AD or asthma after a Bonferroni correction.

After LD clumping and searching in the Phenoscanner database, the remaining SNPs are shown in **Supplementary Tables S5–S16**. After excluding distortion and palindromic ambiguous SNPs, we used the remaining SNPs as final IVs to estimate the causal effect of CVD on AD and asthma. The “leave-one-out” test results and F-statistics are provided in **Supplementary Figures S9**, **S10** and **Supplementary Table S17**. Based on the IVW-FE model, heart failure might be a protective factor against AD (OR=0.0045, 95% CI: 1.1890E-04 - 0.1695, P=0.004). Atherosclerosis (OR=8.7371E-08, 95% CI: 1.8794E-14 - 4.0617E-01, P=0.038) and aortic aneurysm and dissection (OR=1.7367E-07, 95% CI: 3.8390E-14 – 7.8567E-01, P=0.046) might be protective factors for asthma. However, no robust causal effect was detected between all 12 CVD and AD or asthma after the Bonferroni correction (**Figure 3**). The outcomes of other models are displayed in **Supplementary Table S21**. No horizontal pleiotropy or heterogeneity was suggested, as seen in **Supplementary Tables S19**, **S20**. Moreover, the funnel plots, scatter plots, and forest plots for each association pair for casualty are provided in **Supplementary Figures S11–S16**.

## Discussion

This bidirectional MR analysis offers evidence of a robust association between asthma and atrial fibrillation, even after applying the Bonferroni correction. AD exhibits a suggestive connection with essential hypertension. Conversely, heart failure, atherosclerosis, and aortic aneurysm and dissection might reduce the risk of AD and asthma. However, the reverse study did not show robust causality after the Bonferroni correction, indicating that there is only weak evidence to support that CVD causes AD.

To our knowledge, this is the first MR study to determine the bidirectional causal relationship between AD and CVD. A previous MR study established that asthma and atopic dermatitis are causal risk factors for heart failure (*P*=0.03 and 0.01) (25). However, the study could not demonstrate their causal link after the Bonferroni correction. Previous observational studies have produced conflicting conclusions. The presence of AD might be linked to an increased risk of CVD, including atrial fibrillation (5), atherosclerosis (4), heart attack (8), heart failure (8), hypertension (9, 10), stroke (6), and others. Moreover, the co-existence of allergic and CVD may increase the mortality of both diseases (6, 7). However, a decreased risk of CVD and all-cause mortality has also been reported in individuals with allergic rhinitis (11). Observational studies are typically affected by reverse causality or confounding factors, which could be avoided by an MR study.

Regarding the potential mechanism of these two types of diseases, experimental studies suggest that inflammatory and immune responses could be potent mechanisms (3, 4). AD, such as asthma, atopic dermatitis, and rheumatic arthritic, can induce chronic inflammation, which is associated with CVD (4–10). IgE and IgG are essential molecules in allergic reactions (26) associated with atherosclerosis (4). IgE-induced M1 macrophage polarization, foam cell formation, and vascular cell apoptosis contribute to plaque progression (26). Anti-IgE may serve as a protective factor for atherosclerosis, and treatment with an anti-IgE-neutralizing antibody could accelerate atherosclerotic lesion formation (27). High serum concentrations of IgG have been detected in ApoE-/- mice (28), and oxLDL-specific IgG has been observed in human plaques (29), using antibodies that may hinder the rapid regression of atherosclerotic lesions (30). Th2 cells are central to late-phase allergic inflammation, and a high Th2 cell frequency in circulation is associated with a reduced risk of myocardial infarction and stroke (31). Th2-related cytokine interleukin (IL)-4 may be a critical cytokine in early atherosclerosis progression (32), but IL-13 demonstrates a protective effect (33). Active mast cells can induce matrix degradation and apoptosis and recruit inflammatory cells to promote CVD progression (34–36). Apart from the immunological system’s pathophysiological processes, other conditions such as metabolism disorders (37, 38), central obesity (39), and typical lifestyle (40) could also contribute to the potential association between the two diseases. Some observational cohort studies have found a clinical connection between asthma and dyslipidemia, a significant risk factor for CVD (37, 38). Adults with eczema were more likely to experience sleep disturbances and smoke (40). However, our findings partially contradict these observational and experimental studies. We found that asthma is a risk factor for atrial fibrillation, but there was no causality between other AD and CVD. There may be a gap between the hypothesis, experimental studies, and the actual human situation.

This study has several limitations. First, the wide confidence interval around the effect estimate in the reverse study remains compatible with non-inferiority, limiting conclusions about the causal association between two diseases. Second, we used P<5×10^-6^ and 5×10^-5^ as the standard of genome-wide significance to select the variants related to asthma and the 12 kinds of CVD. The relatively small number of SNPs involved, especially in CVD and asthma, might make the SNPs less specific. Moreover, we did not stratify the causal association between AD and CVD by gender, age, BMI, and other factors. However, some studies suggest that these factors may affect the causality. Third, we were unable to detect an association between other AD and CVD due to the limited public genetic data. The complete data could only be obtained for participants of European descent, and we did not verify our conclusion in non-European populations. The instruments identified in European populations may not be suitable for non-European populations. Fourth, a small sample overlap between AD and CVD may lead to bias (41). Within large biobanks with sample sizes larger than 100,000, most two-sample MR methods can be safely used for one-sample MR, even with substantial confounding, particularly the IVW-FE, IVW-RM, weighted median, and weighted mode estimator, which are more robust to pleiotropy than one-sample MR methods (42). Since the UK Biobank is a large dataset with over 300,000 participants, and various compatible methods performing well in one-sample MR applied to our study, we believe our conclusions can be trusted. Furthermore, whole-genome arrays consist of common alleles only, which capture information on common but not rare alleles. However, sometimes low-frequency variants have larger effects than the common ones, although common alleles produce large effect sizes (43). More reliable instruments and larger samples are needed for more precise results.

Despite its limitations, the MR study had several advantages. First, we included variants across the phenotypes of AD and asthma from a recent meta-analysis. We extracted the IVs of the 12 kinds of CVD from the largest GWAS. Second, the bidirectional MR method reduced potential bias from confounding factors and reversed causality, ensuring a clear causal direction in our analysis. Third, we assessed the association between AD and a wide range of CVD, most of which were not previously examined based on genetic instruments. Fourth, it was easy to obtain extensive genetic data from the public genetic dataset using the MR method. The summary statistics can also be applied to individual-level data in terms of statistical power (44).

## Conclusions

In summary, a robust association exists between asthma and atrial fibrillation, indicating that patients with asthma may have an increased risk of atrial fibrillation in European individuals. AD shows a suggestive association with essential hypertension, which attenuates significantly after applying the Bonferroni correction. In the reverse analysis, heart failure, atherosclerosis, and aortic aneurysm and dissection are negatively correlated with AD and asthma. However, the reverse study does not show robust causality after the Bonferroni correction, suggesting weak evidence. Whether AD exerts effects on CVD needs further investigation.

## Data availability statement

The original contributions presented in the study are included in the article/**Supplementary Material**. Further inquiries can be directed to the corresponding authors.

## Author contributions

SW chose the topic. ZW and PH identified genetic variants associated with exposure. SW, HL and PWY completed the subsequent data analysis and article writing. SC, JX and PY provided guidance and assistance throughout the process. All authors contributed to the article and approved the submitted version.
