# Supplementary material for: Exploring the genetic association of allergic diseases with cardiovascular diseases: a bidirectional Mendelian randomization study
Source: Front Immunol. 2023 Jun 2;14:1175890. doi: 10.3389/fimmu.2023.1175890 (PMC10272545; doi:10.3389/fimmu.2023.1175890)
Supplement: Supplementary file 2 [file DataSheet_2.pdf]

Supplementary Figures

Figure S1: Leave-one-out inverse-variance weighted mendelian randomization analyses of allergic disease on different kinds of cardiovascular diseases....1

Figure S2: Leave-one-out inverse-variance weighted mendelian randomization analyses of asthma on different kinds of cardiovascular diseases....2

Figure S3: Funnel plot on allergic disease and different types of cardiovascular diseases....3

Figure S4: Funnel plot on asthma and different types of cardiovascular diseases....4

Figure S5: Scatter plot of the causal effect of allergic disease on different types of cardiovascular diseases....5

Figure S6: Scatter plot of the causal effect of asthma on different types of cardiovascular diseases....6

Figure S7: Forest plots for association of allergic disease with cardiovascular diseases....7

Figure S8: Forest plots for association of asthma with cardiovascular diseases....8

Figure S9: Leave-one-out inverse-variance weighted mendelian randomization analyses of different kinds of cardiovascular diseases on allergic disease....9

Figure S10: Leave-one-out inverse-variance weighted mendelian randomization analyses of different kinds of cardiovascular diseases on asthma....10

Figure S11: Funnel plot on different types of cardiovascular diseases and allergic disease...11

Figure S12: Funnel plot on different types of cardiovascular diseases and asthma....12

Figure S13: Scatter plot of the causal effect of different types of cardiovascular diseases on allergic disease....13

Figure S14: Scatter plot of the causal effect of different types of cardiovascular diseases on asthma....14

Figure S15: Forest plots for association of cardiovascular diseases with allergic disease....15

Figure S16: Forest plots for association of cardiovascular diseases with asthma....16

Supplementary Figure S1

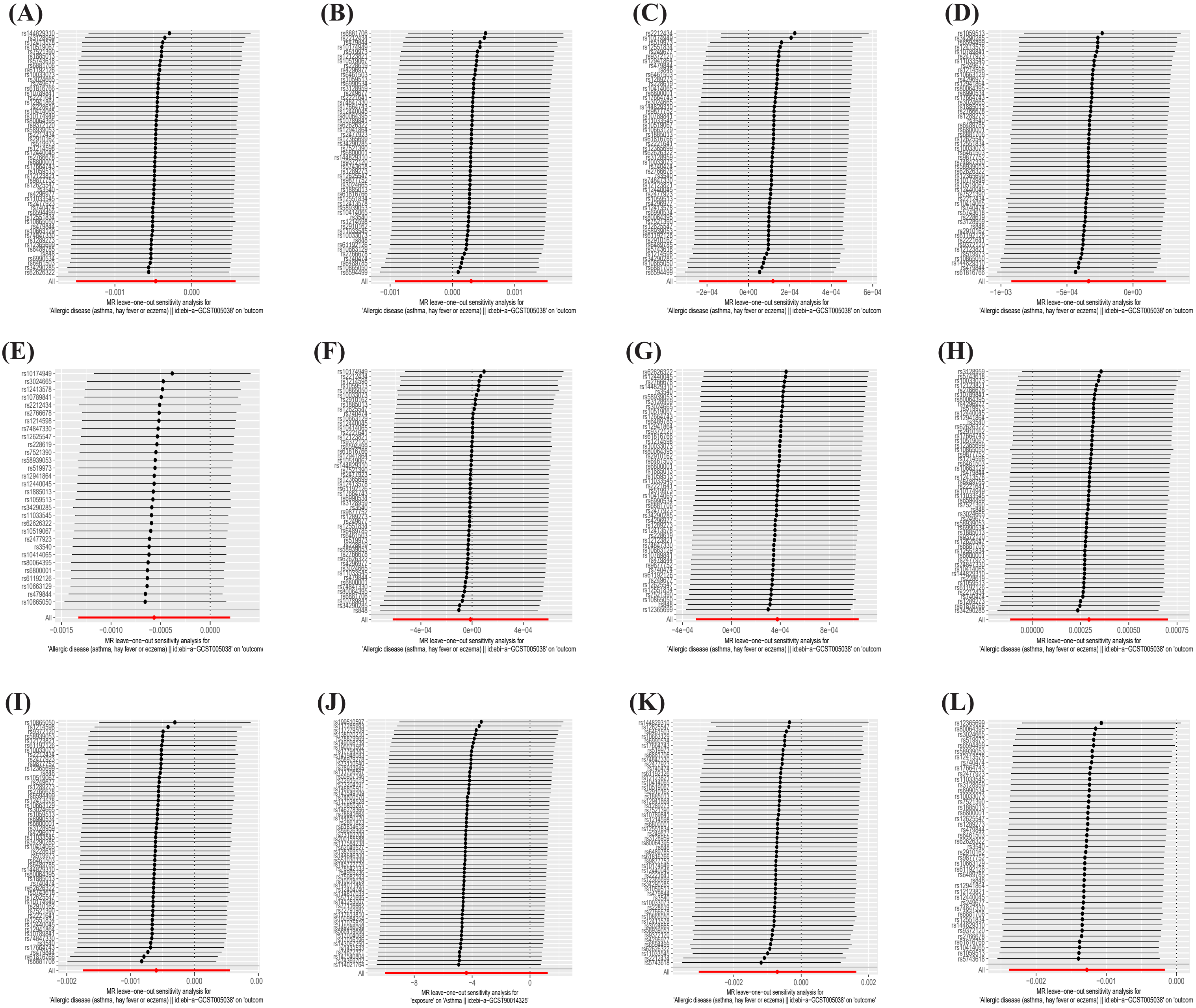

Figure S1: Leave-one-out inverse-variance weighted mendelian randomization analyses of allergic disease (asthma, hay fever or eczema) on different kinds of cardiovascular diseases: (A) Heart arrhythmia; (B) Atrial fibrillation; (C) Supraventricular tachycardia; (D) Atherosclerosis; (E) Aortic aneurysm and dissection; (F) Stroke; (G) Peripheral vascular disease; (H) Cardiomyopathy; (I) Heart valve problem; (J) Heart failure; (K) Myocardial infarction; (L) Essential hypertension.

Supplementary Figure S2

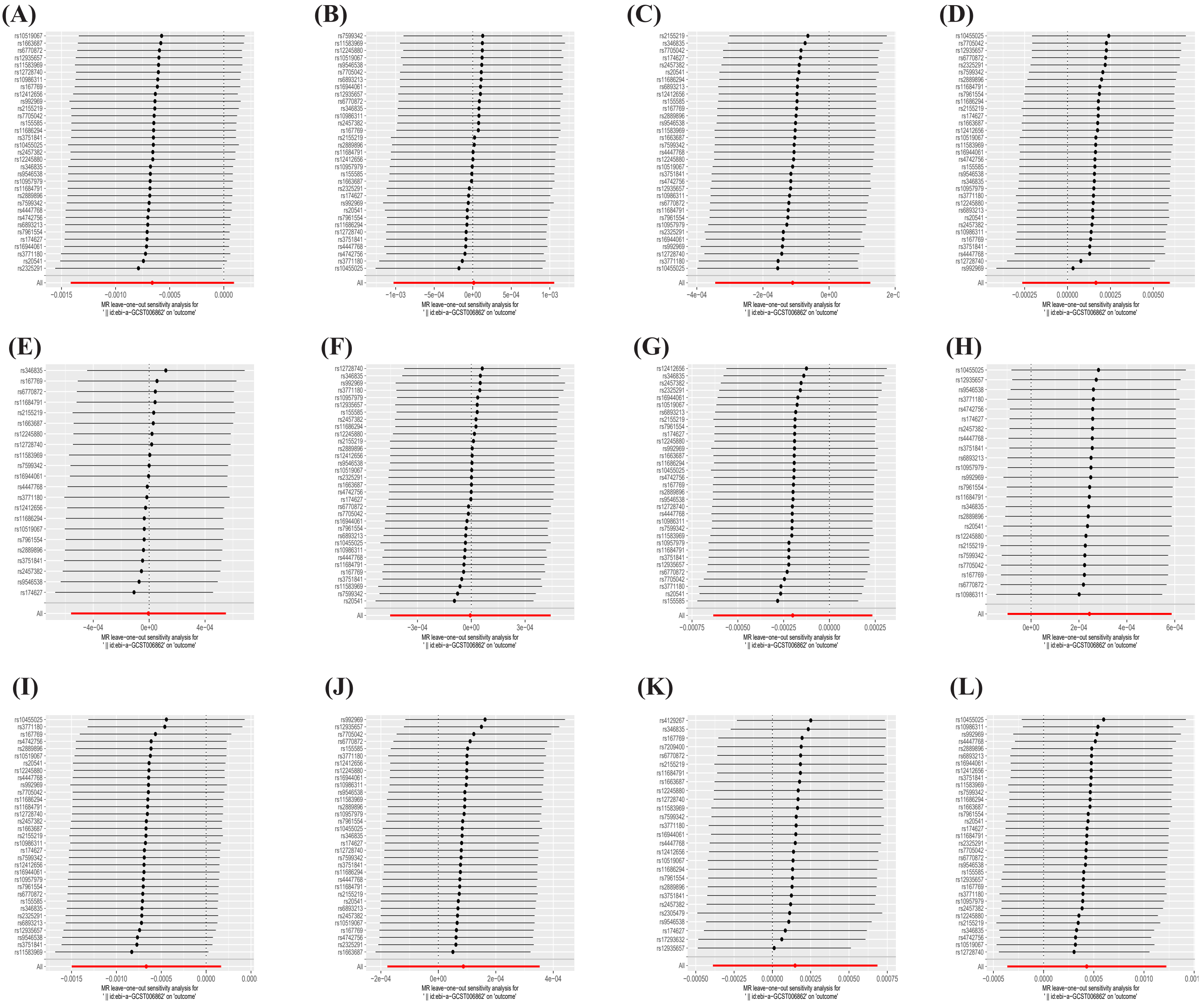

Figure S2: Leave-one-out inverse-variance weighted mendelian randomization analyses of asthma on different kinds of cardiovascular diseases: (A) Heart arrhythmia; (B) Atrial fibrillation; (C) Supraventricular tachycardia; (D) Atherosclerosis; (E) Aortic aneurysm and dissection; (F) Stroke; (G) Peripheral vascular disease; (H) Cardiomyopathy; (I) Heart valve problem; (J) Heart failure; (K) Myocardial infarction; (L) Essential hypertension.

Supplementary Figure S3

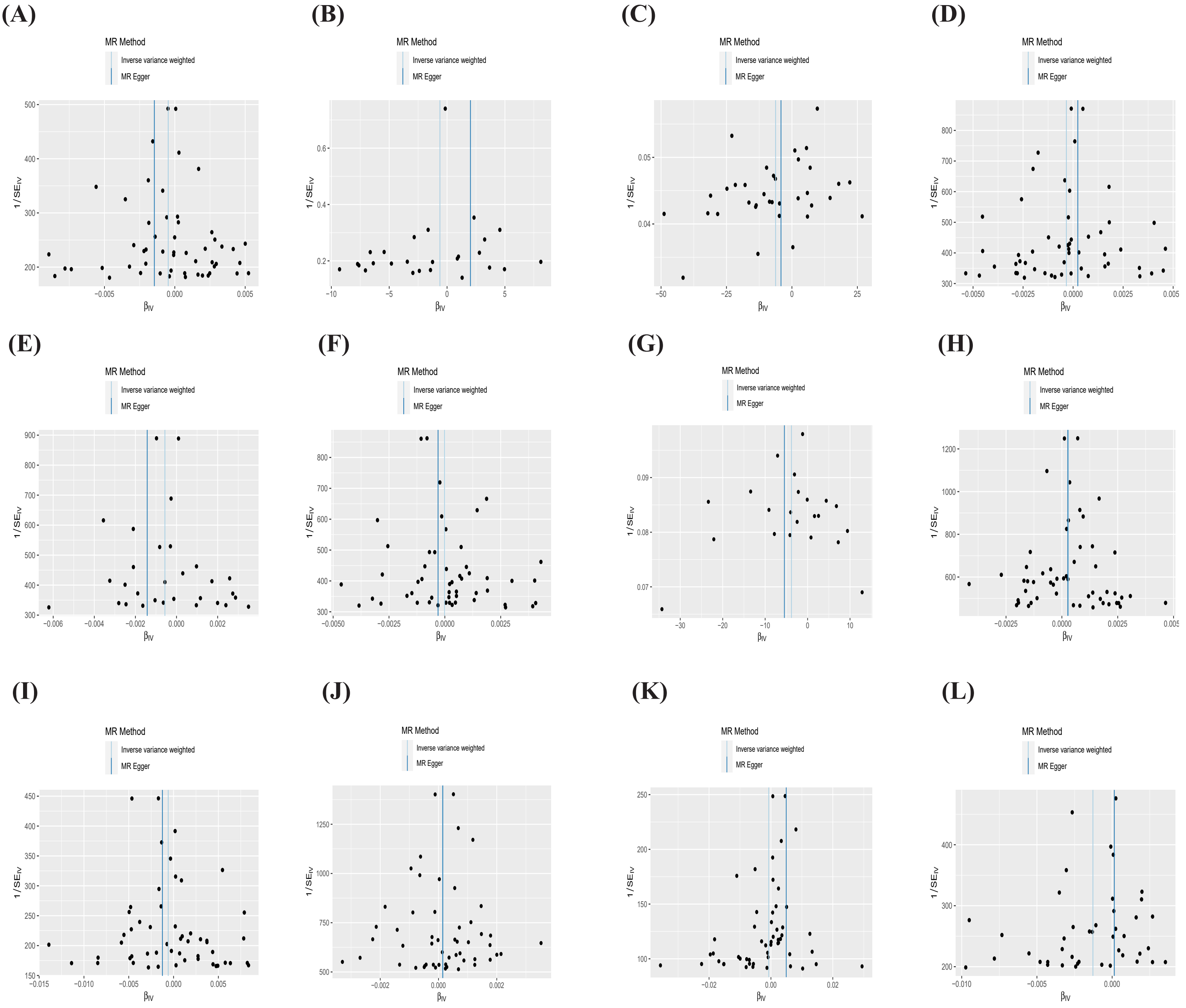

Figure S3: Funnel plot on allergic disease (asthma, hay fever and eczema) and different types of cardiovascular diseases : (A) Heart arrhythmia; (B) Atrial fibrillation; (C) Supraventricular tachycardia; (D) Atherosclerosis; (E) Aortic aneurysm and dissection; (F) Stroke; (G) Peripheral vascular disease; (H) Cardiomyopathy; (I) Heart valve problem; (J) Heart failure; (K) Myocardial infarction; (L) Essential hypertension.

Supplementary Figure S4

(A)

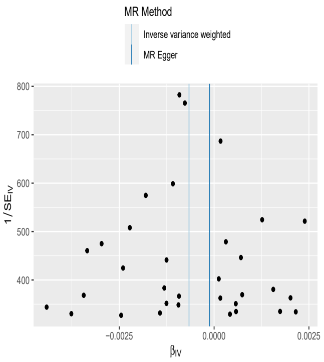

(B)

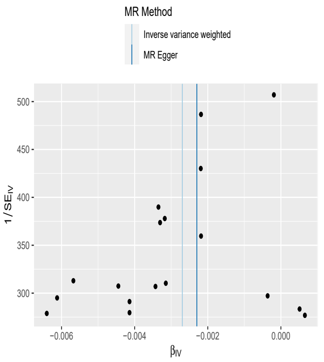

(C)

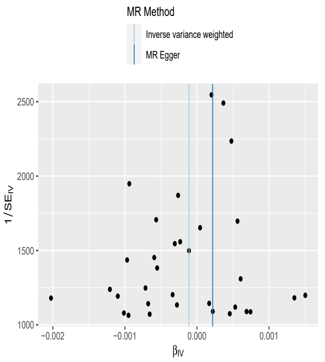

(D)

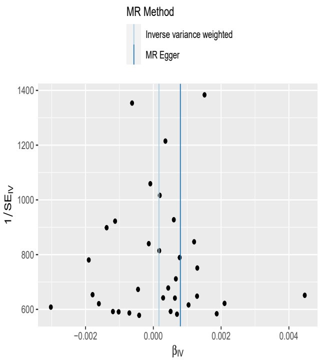

(E)

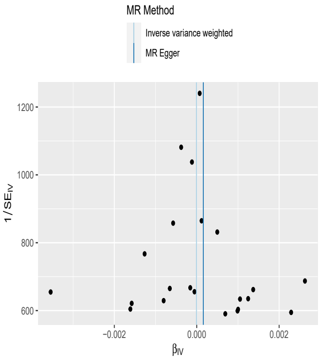

(F)

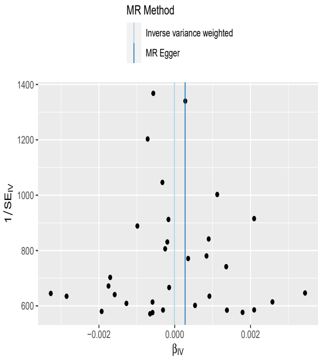

(G)

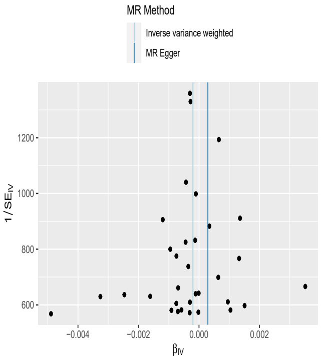

(H)

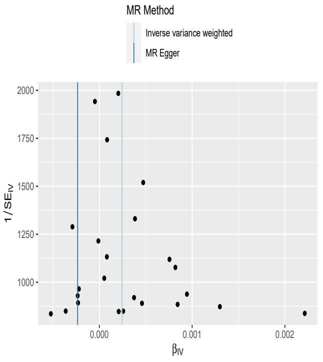

(I)

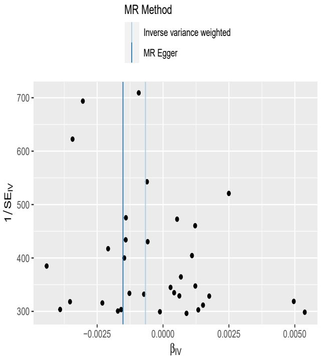

(J)

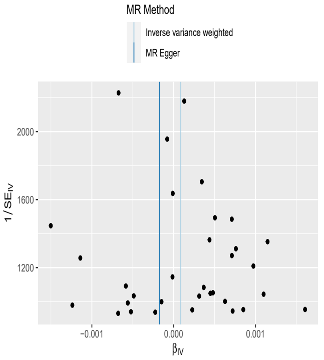

(K)

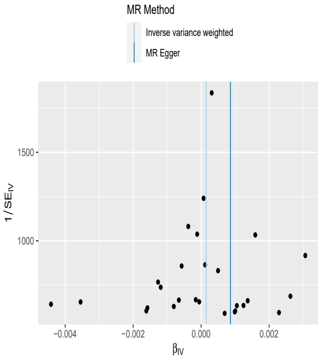

(L)

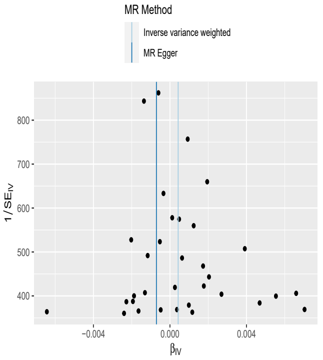

Figure S4: Funnel plot on asthma and different types of cardiovascular diseases : (A) Heart arrhythmia; (B) Atrial fibrillation; (C) Supraventricular tachycardia; (D) Atherosclerosis; (E) Aortic aneurysm and dissection; (F) Stroke; (G) Peripheral vascular disease; (H) Cardiomyopathy; (I) Heart valve problem; (J) Heart failure; (K) Myocardial infarction; (L) Essential hypertension.

Supplementary Figure S5

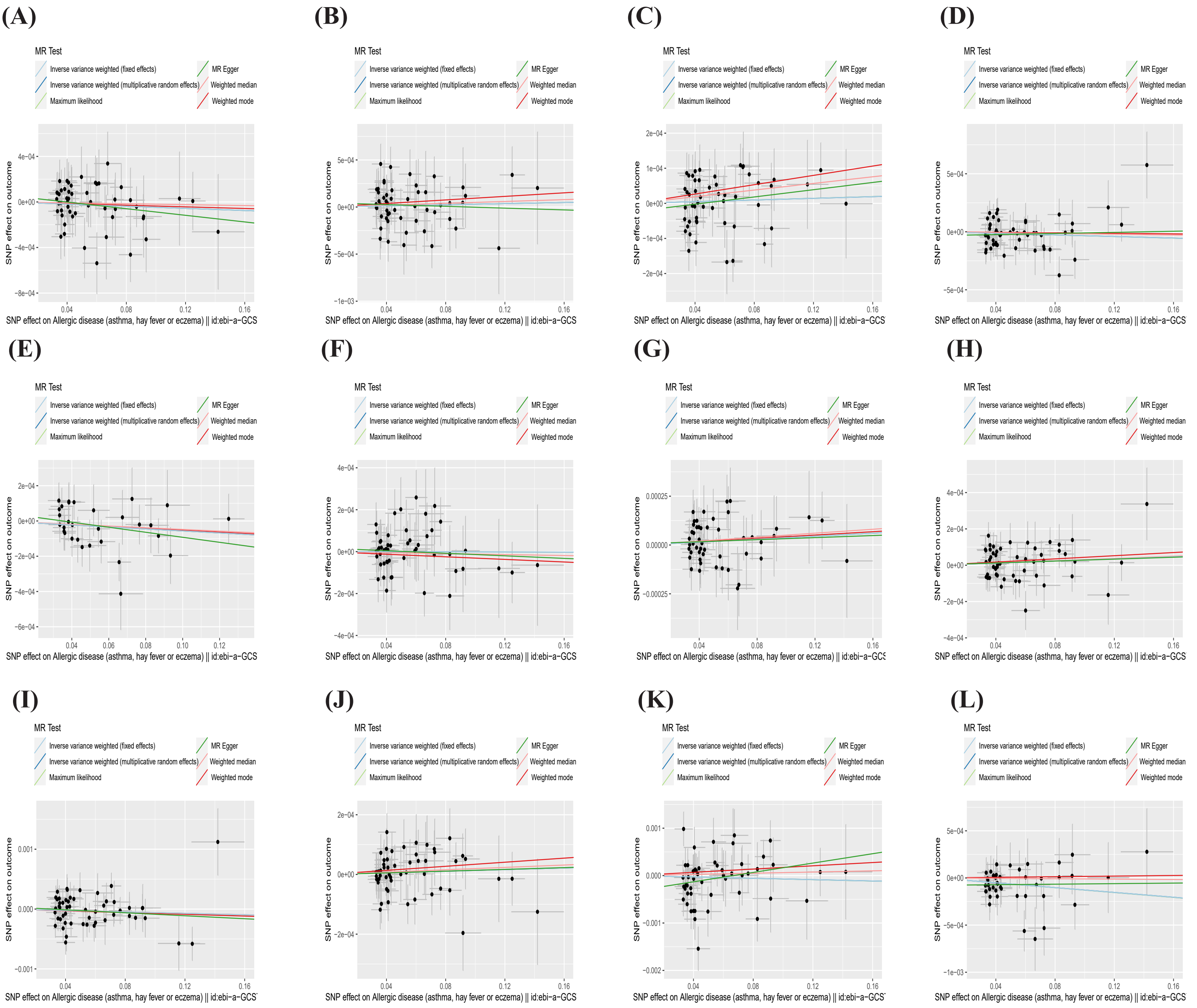

Figure S5: Scatter plot of the causal effect of allergic disease (asthma, hay fever and eczema) on different types of cardiovascular diseases: (A) Heart arrhythmia; (B) Atrial fibrillation; (C) Supraventricular tachycardia; (D) Atherosclerosis; (E) Aortic aneurysm and dissection; (F) Stroke; (G) Peripheral vascular disease; (H) Cardiomyopathy; (I) Heart valve problem; (J) Heart failure; (K) Myocardial infarction; (L) Essential hypertension **with the slope of each line corresponding to the estimated causal effect per method**.

Supplementary Figure S6

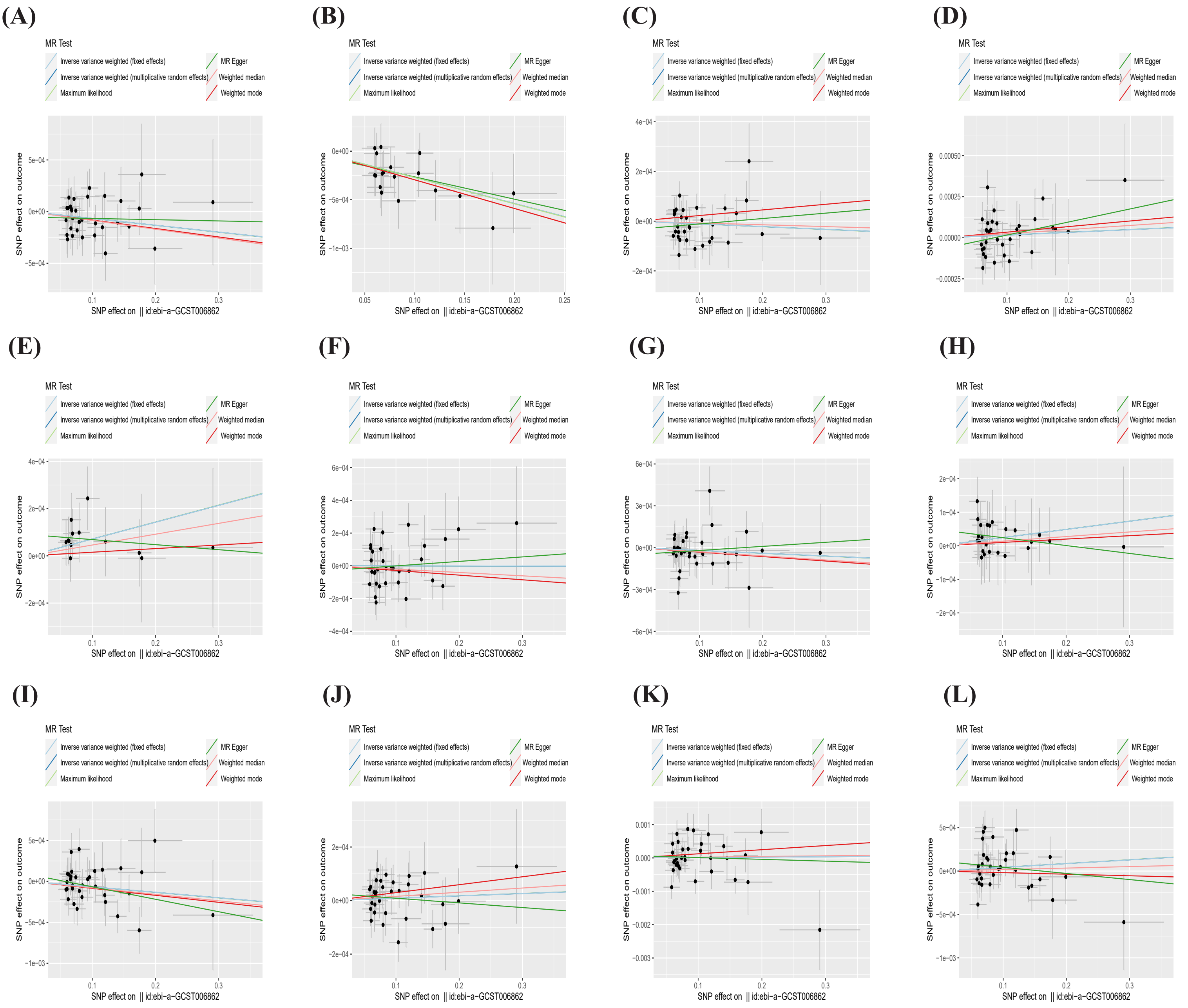

Figure S6: Scatter plot of the causal effect of asthma on different types of cardiovascular diseases: (A) Heart arrhythmia; (B) Atrial fibrillation; (C) Supraventricular tachycardia; (D) Atherosclerosis; (E) Aortic aneurysm and dissection; (F) Stroke; (G) Peripheral vascular disease; (H) Cardiomyopathy; (I) Heart valve problem; (J) Heart failure; (K) Myocardial infarction; (L) Essential hypertension **with the slope of each line corresponding to the estimated causal effect per method**.

Supplementary Figure S7

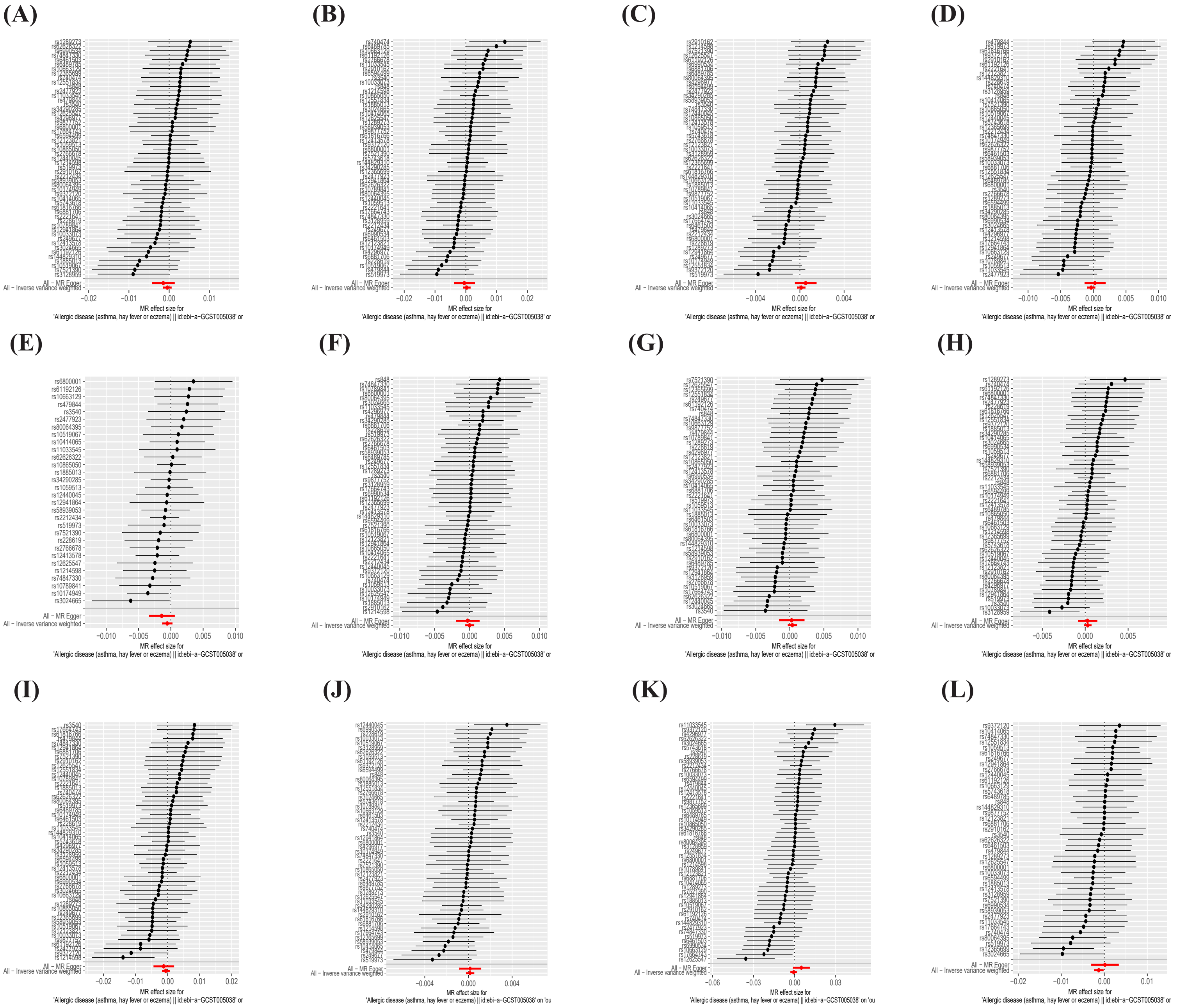

Figure S7: Forest plots for association of allergic disease (asthma, hay fever and eczema) with cardiovascular diseases: A) Heart arrhythmia; (B) Atrial fibrillation; (C) Supraventricular tachycardia; (D) Atherosclerosis; (E) Aortic aneurysm and dissection; (F) Stroke; (G) Peripheral vascular disease; (H) Cardiomyopathy; (I) Heart valve problem; (J) Heart failure; (K) Myocardial infarction; (L) Essential hypertension. **Data are expressed as beta values with 95% CI.**

Supplementary Figure S8

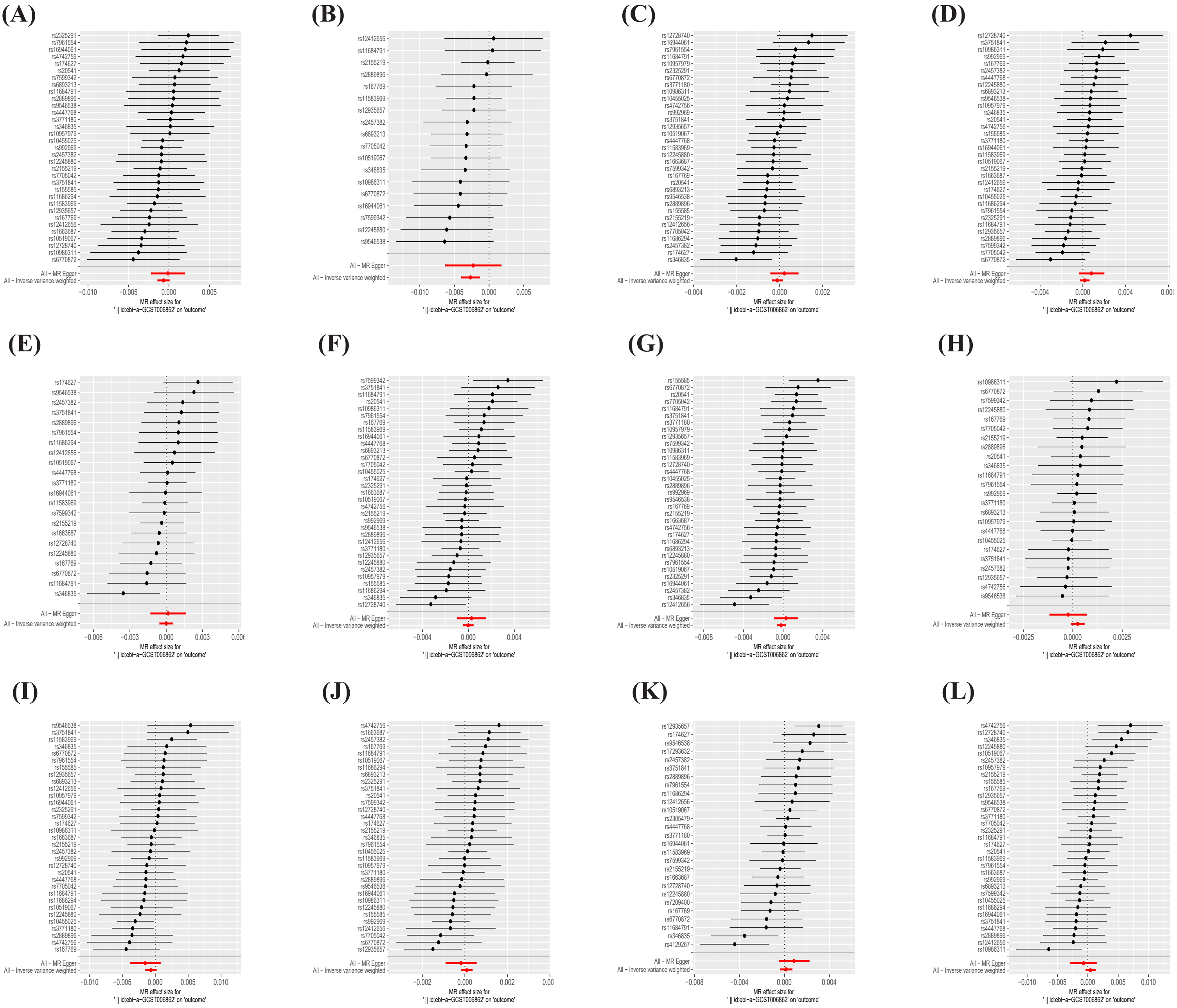

Figure S8: Forest plots for association of asthma with cardiovascular diseases: A) Heart arrhythmia; (B) Atrial fibrillation; (C) Supraventricular tachycardia; (D) Atherosclerosis; (E) Aortic aneurysm and dissection; (F) Stroke; (G) Peripheral vascular disease; (H) Cardiomyopathy; (I) Heart valve problem; (J) Heart failure; (K) Myocardial infarction； (L) Essential hypertension. **Data are expressed as beta values with 95% CI.**

Supplementary Figure S9

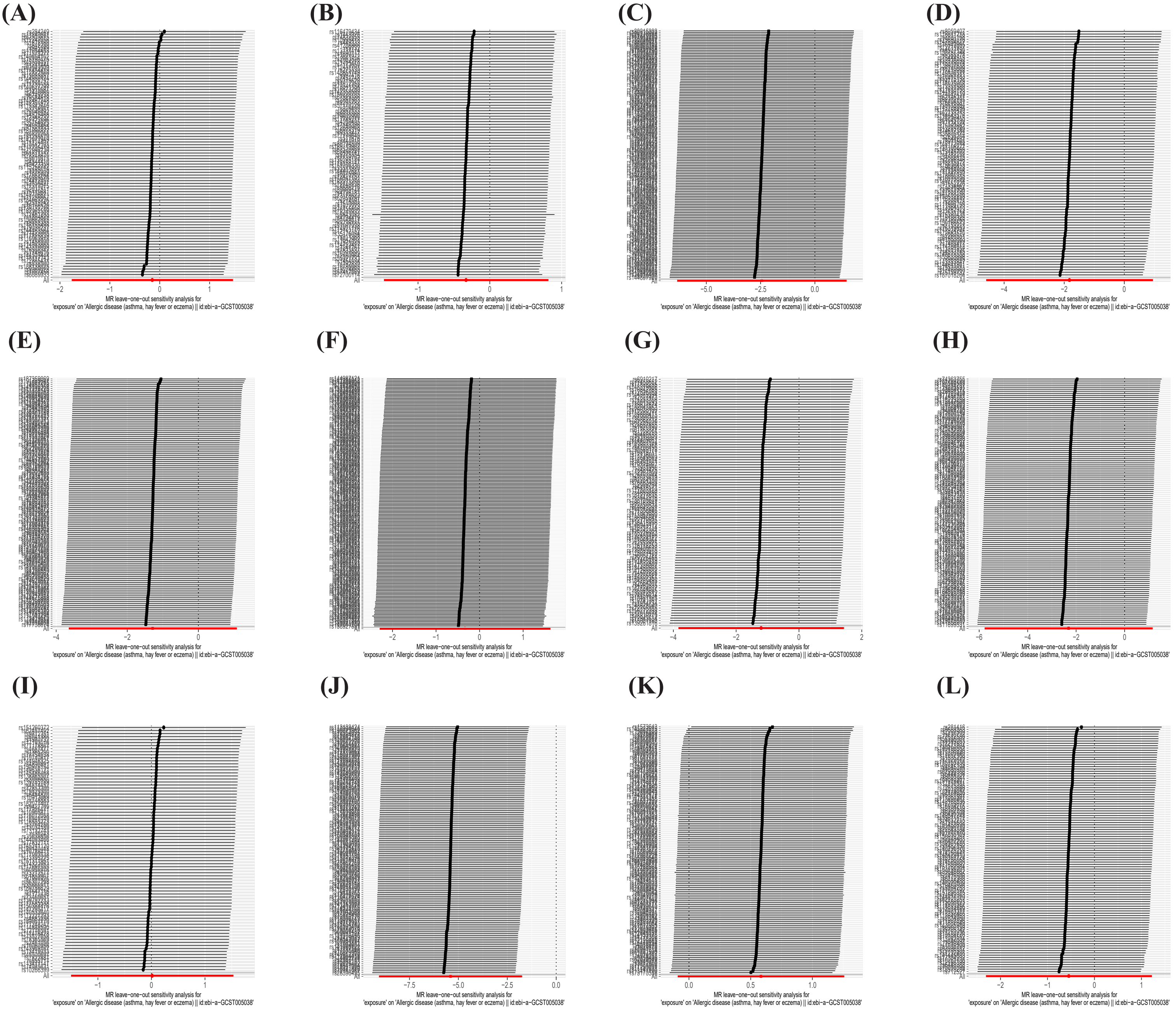

Figure S9: Leave-one-out inverse-variance weighted mendelian randomization analyses of different kinds of cardiovascular diseases: (A) Heart arrhythmia; (B) Atrial fibrillation; (C) Supraventricular tachycardia; (D) Atherosclerosis; (E) Aortic aneurysm and dissection; (F) Stroke; (G) Peripheral vascular disease; (H) Cardiomyopathy; (I) Heart valve problem; (J) Heart failure; (K) Myocardial infarction; (L) Essential hypertension **on allergic diseases (asthme, hay fever and eczema).**

Supplementary Figure S10

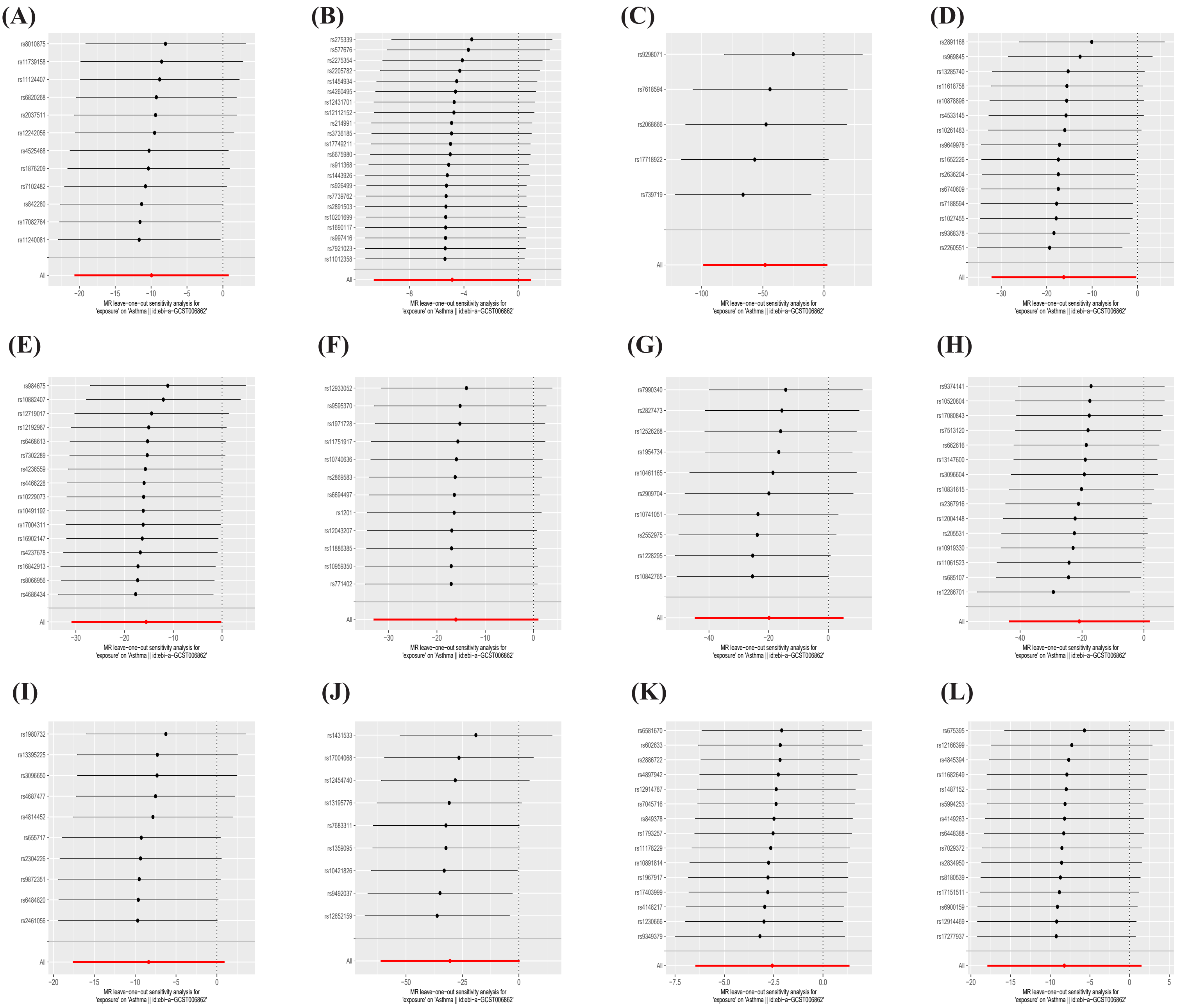

Figure S10: Leave-one-out inverse-variance weighted mendelian randomization analyses of different kinds of cardiovascular diseases: (A) Heart arrhythmia; (B) Atrial fibrillation; (C) Supraventricular tachycardia; (D) Atherosclerosis; (E) Aortic aneurysm and dissection; (F) Stroke; (G) Peripheral vascular disease; (H) Cardiomyopathy; (I) Heart valve problem; (J) Heart failure; (K) Myocardial infarction; (L) Essential hypertension **on asthma**.

Supplementary Figure S11

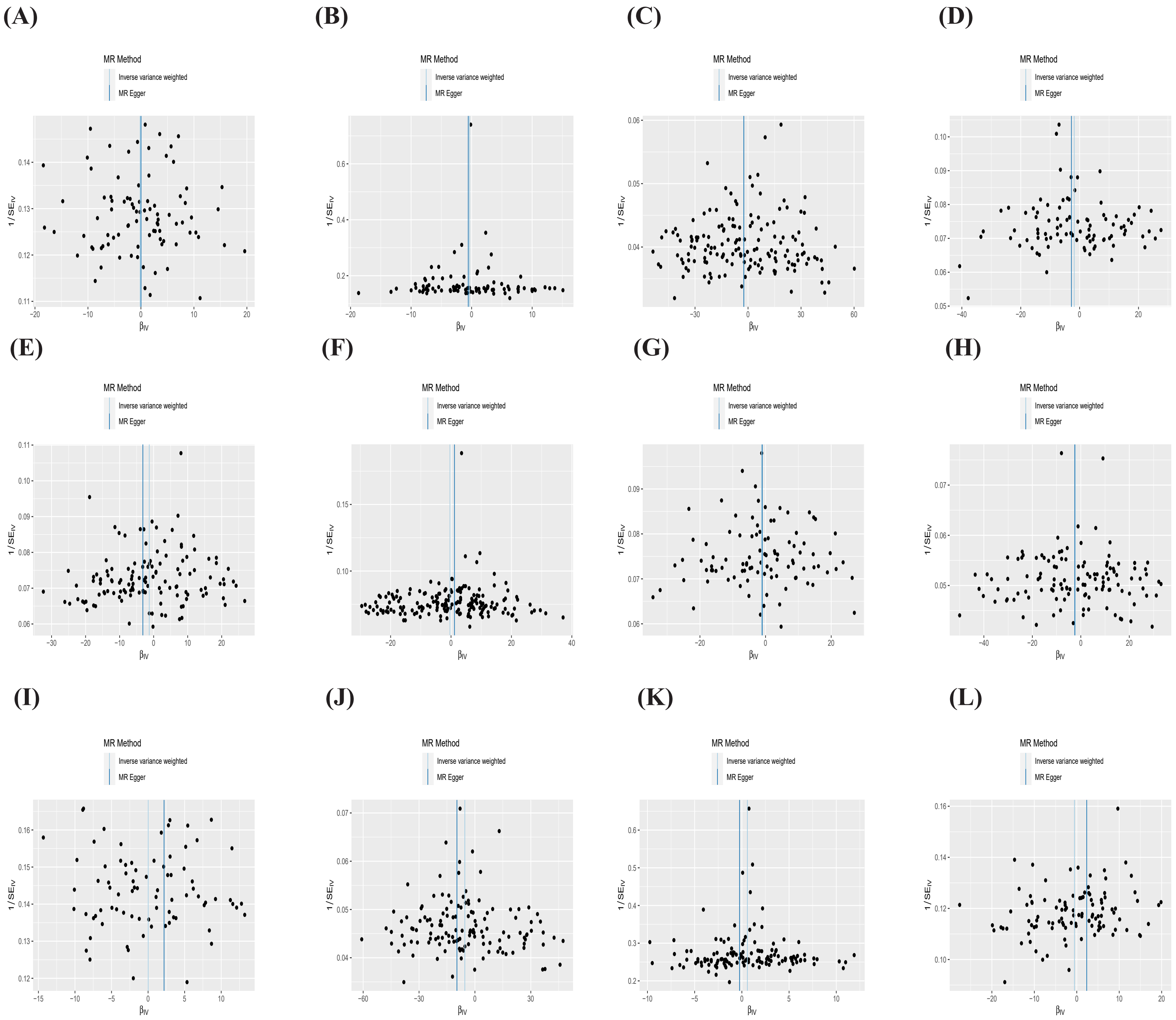

Figure S11: Funnel plot on different types of cardiovascular diseases : (A) Heart arrhythmia; (B) Atrial fibrillation; (C) Supraventricular tachycardia; (D) Atherosclerosis; (E) Aortic aneurysm and dissection; (F) Stroke; (G) Peripheral vascular disease; (H) Cardiomyopathy; (I) Heart valve problem; (J) Heart failure; (K) Myocardial infarction; (L) Essential hypertension **and allergic disease (asthma, hay fever and eczema)**.

Supplementary Figure S12

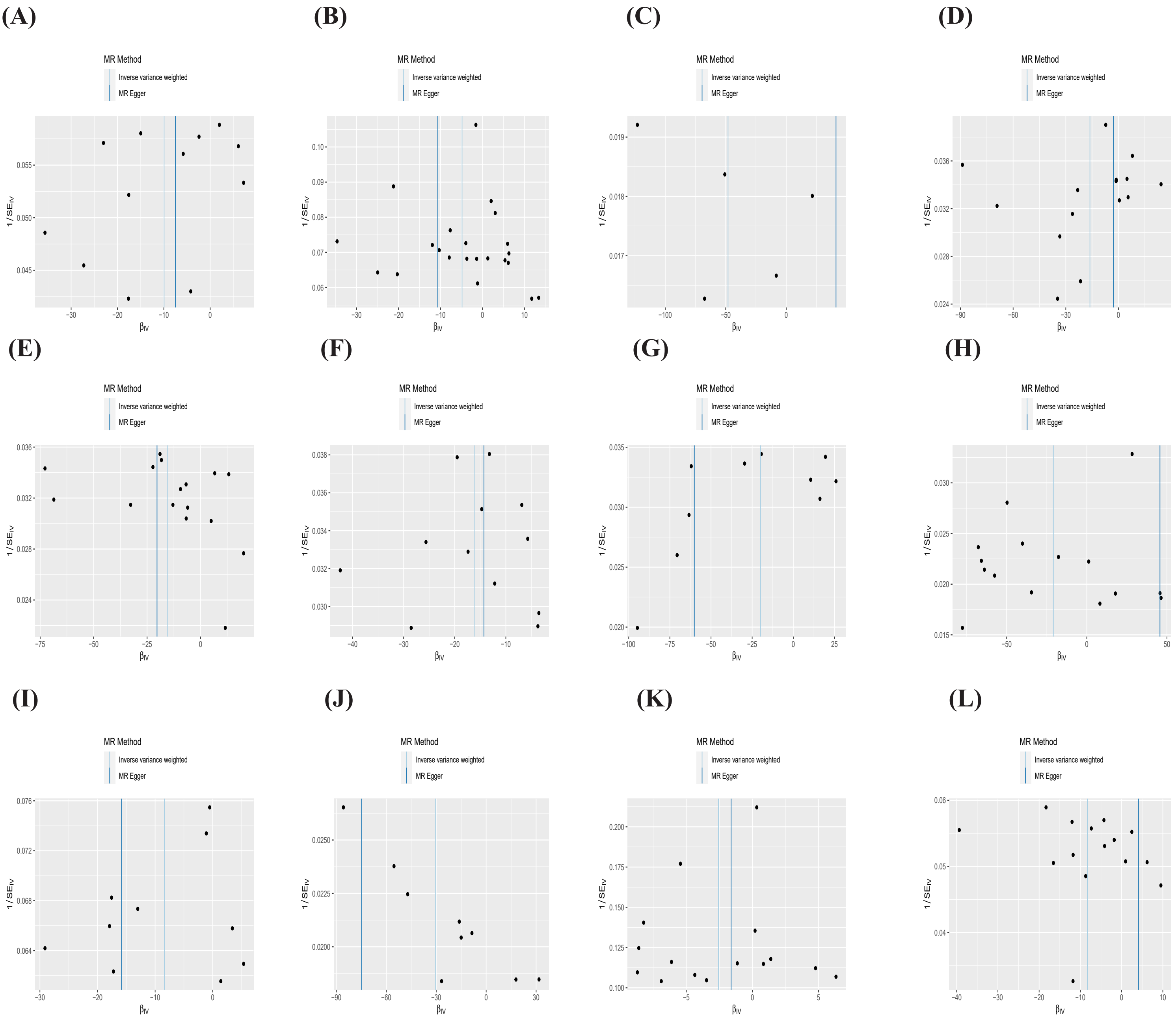

Figure S12: Funnel plot on different types of cardiovascular diseases : (A) Heart arrhythmia; (B) Atrial fibrillation; (C) Supraventricular tachycardia; (D) Atherosclerosis; (E) Aortic aneurysm and dissection; (F) Stroke; (G) Peripheral vascular disease; (H) Cardiomyopathy; (I) Heart valve problem; (J) Heart failure; (K) Myocardial infarction; (L) Essential hypertension **and asthma**.

Supplementary Figure S13

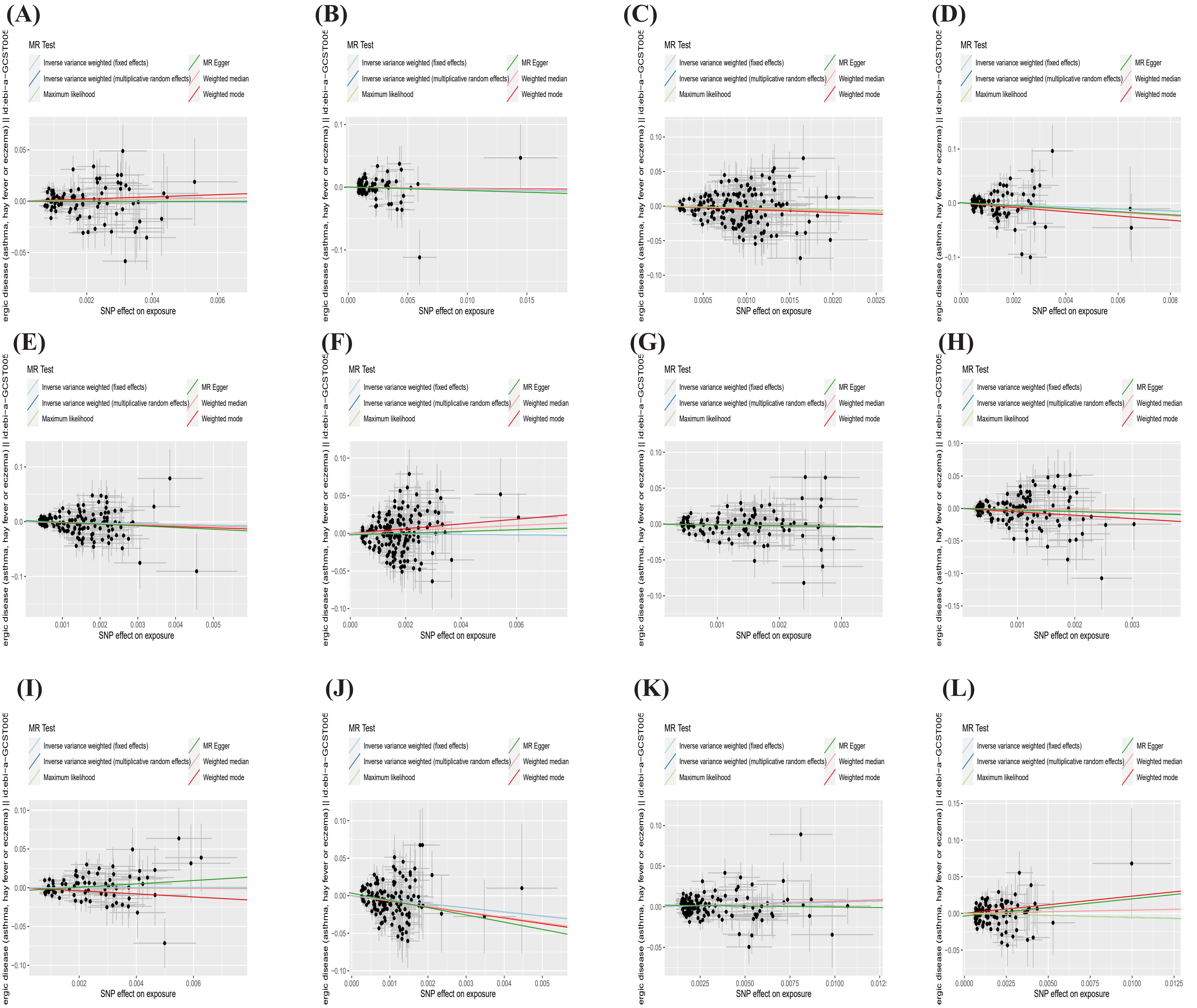

Figure S13: Scatter plot of the causal effect of different types of cardiovascular diseases: (A) Heart arrhythmia; (B) Atrial fibrillation; (C) Supraventricular tachycardia; (D) Atherosclerosis; (E) Aortic aneurysm and dissection; (F) Stroke; (G) Peripheral vascular disease; (H) Cardiomyopathy; (I) Heart valve problem; (J) Heart failure; (K) Myocardial infarction; (L) Essential hypertension **on allergic disease (asthma, hay fever and eczema) with the slope of each line corresponding to the estimated causal effect per method**.

Supplementary Figure S14

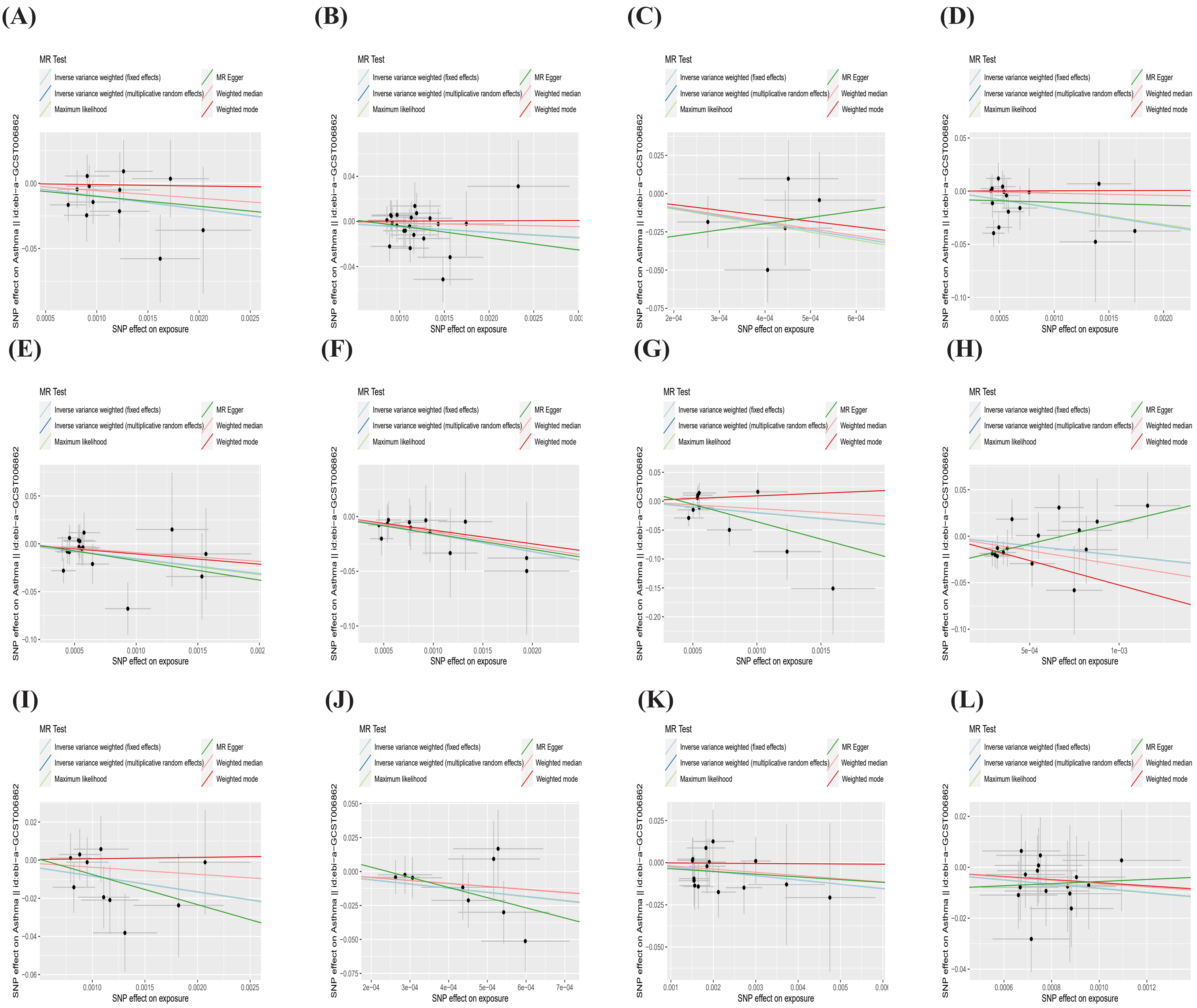

Figure S14: Scatter plot of the causal effect of different types of cardiovascular diseases: (A) Heart arrhythmia; (B) Atrial fibrillation; (C) Supraventricular tachycardia; (D) Atherosclerosis; (E) Aortic aneurysm and dissection; (F) Stroke; (G) Peripheral vascular disease; (H) Cardiomyopathy; (I) Heart valve problem; (J) Heart failure; (K) Myocardial infarction; (L) Essential hypertension **on asthma with the slope of each line corresponding to the estimated causal effect per method**.

Supplementary Figure S15

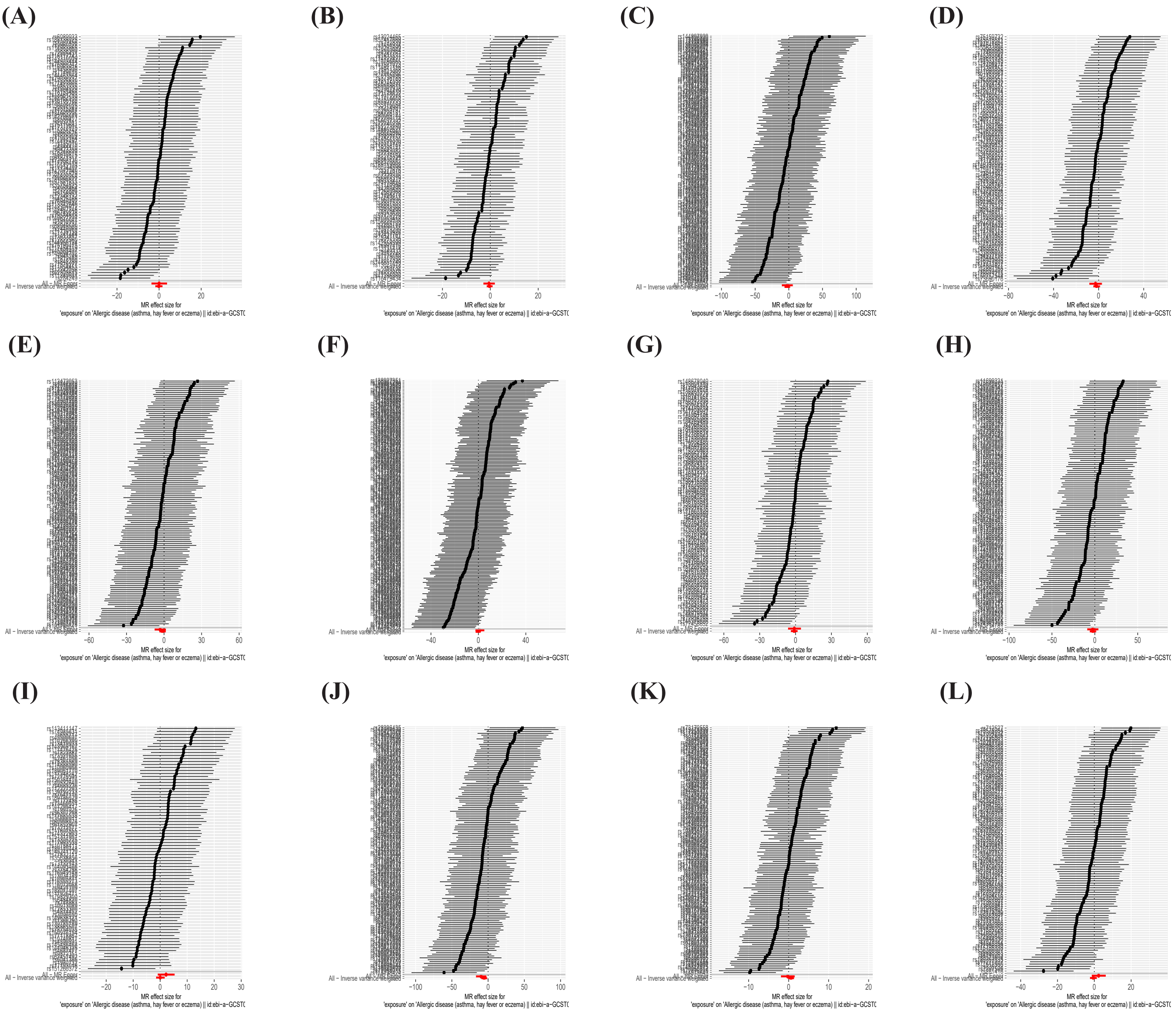

Figure S15: Forest plots for association of cardiovascular diseases: A) Heart arrhythmia; (B) Atrial fibrillation; (C) Supraventricular tachycardia; (D) Atherosclerosis; (E) Aortic aneurysm and dissection; (F) Stroke; (G) Peripheral vascular disease; (H) Cardiomyopathy; (I) Heart valve problem; (J) Heart failure; (K) Myocardial infarction; (L) Essential hypertension **with allergic disease (asthma, hay fever and eczema). Data are expressed as beta values with 95% CI.**

Supplementary Figure S16

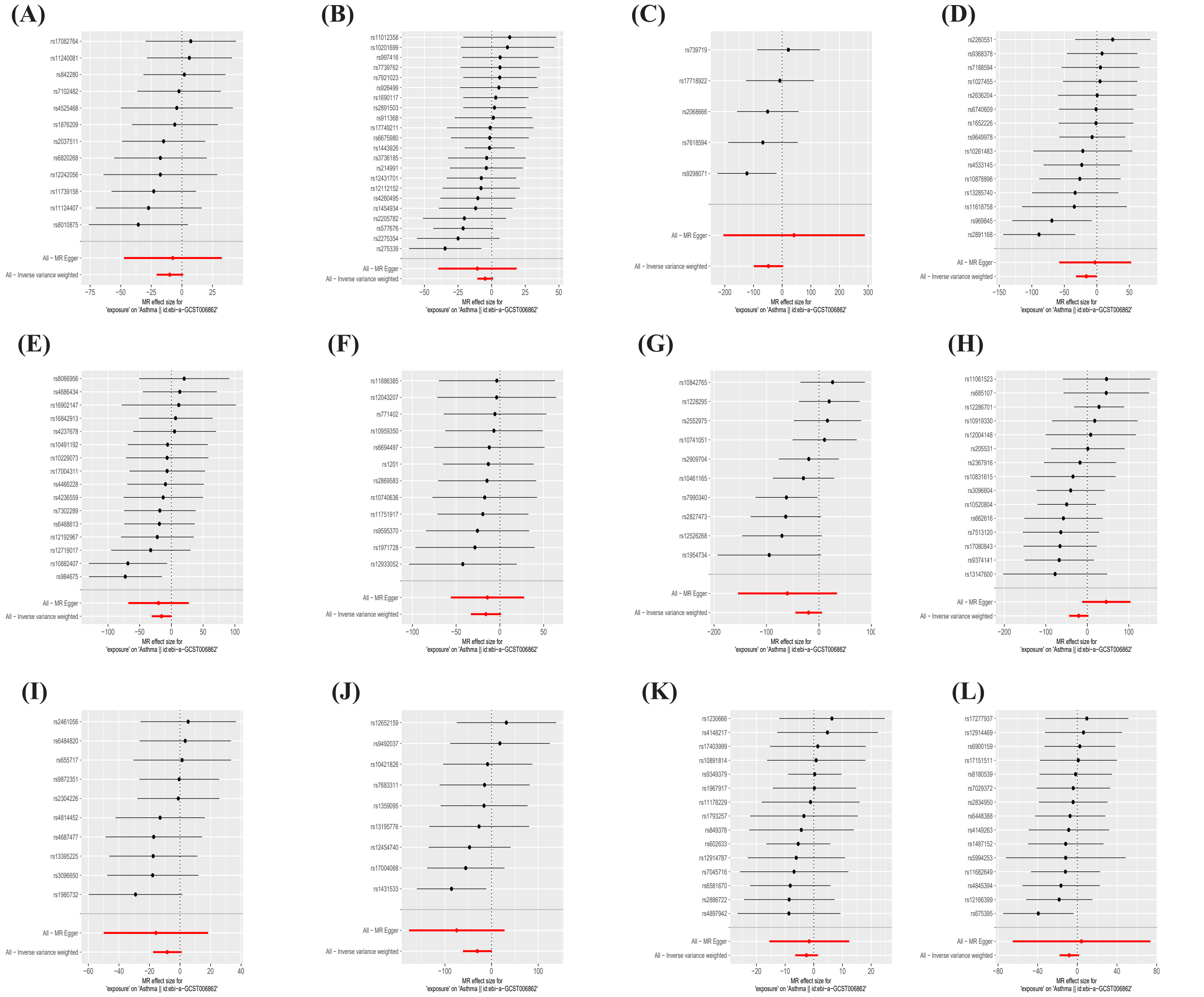

Figure S16: Forest plots for association of cardiovascular diseases: A) Heart arrhythmia; (B) Atrial fibrillation; (C) Supraventricular tachycardia; (D) Atherosclerosis; (E) Aortic aneurysm and dissection; (F) Stroke; (G) Peripheral vascular disease; (H) Cardiomyopathy; (I) Heart valve problem; (J) Heart failure; (K) Myocardial infarction; (L) Essential hypertension **with asthma. Data are expressed as beta values with 95% CI.**
